# Associations between polymorphisms in the IL-4 gene and renal cell carcinoma in Chinese Han population

**DOI:** 10.18632/oncotarget.18427

**Published:** 2017-06-09

**Authors:** Hao Rong, Xue He, Li Wang, Yongjun He, Longli Kang, Tianbo Jin

**Affiliations:** ^1^ Key Laboratory of Molecular Mechanism and Intervention Research for Plateau Diseases of Tibet Autonomous Region, School of Medicine, Xizang Minzu University, Xianyang, Shaanxi 712082, China; ^2^ Key Laboratory of High Altitude Environment and Genes Related to Diseases of Tibet Autonomous Region, School of Medicine, Xizang Minzu University, Xianyang, Shaanxi 712082, China; ^3^ Key Laboratory for Basic Life Science Research of Tibet Autonomous Region, School of Medicine, Xizang Minzu University, Xianyang, Shaanxi 712082, China

**Keywords:** renal cell carcinoma, IL4, Han Chinese, gene polymorphism

## Abstract

Renal cell carcinoma (RCC) is considered to be a kind of cytokine reactive tumor. The research has been suggested that the host immune system can regulate the clinical course of RCC. Therefore, cytokine gene polymorphisms in RCC patients were analyzed was necessary. Our study is purpose to analyzing the interleukin-4(IL-4) polymorphisms associated with RCC risk from Han Chinese population. IL-4 genetic polymorphisms were genotyped using Massarray technology from a total of 291RCC and 463 controls. Unconditional logistic regression analysis was performed to analyze their relationship with risk of RCC. A significant association was found between the rs2243250 “C” allel and decreased risk of RCC (OR=0.75, 95%CI=0.59-0.96, P=0.02). Stratified analysis based on the age, gender, smoking status, drinking status revealed no significant association with RCC in age>55, female, smoking and nodrinking. However, for age<55 group (rs2243250, rs2243267, rs2243270), male group (rs2243250), nonsmoking group (rs2227284), and drinking group (rs2243250, rs2227284, rs2243267, rs2243270) polymorphisms were found obviously associated with RCC. The haplotype analyses showed that the haplotype have a significant decreased risk of RCC in the rs2243250/rs2227284/rs2243267/rs2243270/rs2243283/rs2243289 (CGGACA) (Total, OR=0.73, 95%CI=0.54-0.98, P=0.034; Male, OR=0.59, 95%CI=0.39-0.90, P=0.014). Therefore, the present study suggests that IL-4 may be a candidate gene for assessing the risk of RCC.

## INTRODUCTION

Renal cell carcinoma (RCC) is the most common malignancy of the kidney, the frequency of which is increasing in both, men and women [1]. RCC has a worldwide incidence. It is reported that it is more common in men than women [2]. The exact causes of renal cell carcinoma have not been identified yet, but the evidence from clinical trials and medical experience built up over time reveals a strong connection between several risk factors such as gender, age and smoking [3] and alcohol intake [4] are among other lifestyle factors [5] that are known to increase the risk of RCC.

Genetic and hereditary conditions also increase the risk of RCC. The potential role of immune response in the risk of RCC has been provided [6]. Cytokines participate in mediating many of the effect or phases of immune and inflammatory responses. Recently, genetic polymorphisms potentially affecting production levels of certain cytokines may be important determinants of disease risk, severity or protection for several conditions in which the immune system plays significant roles, such as malignancies. Eventually, many studies have reported that there is an association between cytokine gene polymorphisms and the development of certain infectious diseases, allergies, autoimmune disorders and cancers. In some studies, these genetic polymorphisms are shown to affect to overall expression and secretion of cytokines both *in vitro* and sporadically *in vivo* systems [9]. Interleukin (IL)-4 is produced by activated T lymphocytes and associated with the similar immune responses [7]. IL4 is a multifunctional cytokine, which binds to IL-4 receptor (IL-4R) to mediate the differentiation of antigen stimulated T cells [8]. IL4 might be proved useful as antitumoral and anti-inflammatory agents [9].

The aim of the present study was to investigate whether there are associations between cytokine gene polymorphism and the risk of RCC development. We used IL-4 gene polymorphisms with the RCC association risk in Chinese Han populated samples.

## RESULTS

Table 1 showed the characteristics, there was no significant difference in age, sex, BMI, smoking status between the case group and the control group (P > 0.05), however, difference was statistically significant in drinking status (P < 0.05). Table 2 showed the allele and genotype frequencies of IL-4. The control group followed Hardy-Weinberg equilibrium (P > 0.05). For rs2243250 individuals with “C” allel had slight significant decreased risk of RCC (OR = 0.75, 95%CI = 0.59-0.96, P = 0.02). The results of the various genetic models are displayed in Table 3. The C allele tends to be associated with the decreased risk of RCC in Log-additive model (OR = 0.78, 95%CI = 0.61-0.99, P = 0.043).

**Table 1:** Characteristics of RCC patients and control participants

Variable		Case	Control	P
Total		291	463	
Age(Mean±SD)		56.88±11.66	50.65±11.79	0.273
BMI(Mean±SD)		23.96±2.90	23.79±3.77	0.098
Gender				
	Female	99	198	0.017
	Male	192	265	
Smoking status				
	Smoking	120	169	0.193
	Nosmoking	171	294	
Drinking status				
	Drinking	238	289	<0.05
	Nodrinking	53	174	

**Table 2:** Basic information of candidate SNPs in this study

SNPs	Gene	Band	Allele(A/B)	MAF(case)	MAF(control)	HWE	P	OR(95%CI)
rs2243250	IL4	5q31.1	C/T	0.196	0.245	0.131	0.020	0.75
								(0.59-0.96)
rs2227284	IL4	5q31.1	G/T	0.139	0.172	0.1	0.073	0.78
								(0.59 -1.02)
rs2243267	IL4	5q31.1	G/C	0.199	0.24	0.253	0.053	0.79
								(0.62 -1.00)
rs2243270	IL4	5q31.1	A/G	0.199	0.24	0.253	0.053	0.79
								(0.62-1.00)
rs2243283	IL4	5q31.1	G/C	0.161	0.185	0.120	0.204	0.84
								(0.65-1.10)
rs2243289	IL4	5q31.1	A/G	0.201	0.234	0.244	0.107	0.82
								(0.64-1.04)

OR: odds ratio; 95% CI: 95% confidence interval; HWE p ≤ 0.05 is excluded; MAF: minor allele frequency; Allele (A/B) A: minor allele.

**Table 3:** Association between rs2243250 genetic model and RCC risk in total

Model	Genotype	Control	Case	OR (95% CI)	P-value
Codominant	T/T	270 (58.3%)	188 (64.6%)	1	0.12
	C/T	159 (34.3%)	90 (30.9%)	0.81 (0.59-1.12)	
	C/C	34 (7.3%)	13 (4.5%)	0.55 (0.28-1.07)	
Dominant	T/T	270 (58.3%)	188 (64.6%)	1	0.084
	C/T-C/C	193 (41.7%)	103 (35.4%)	0.77 (0.57-1.04)	
Recessive	T/T-C/T	429 (92.7%)	278 (95.5%)	1	0.1
	C/C	34 (7.3%)	13 (4.5%)	0.59 (0.31-1.14)	
Log-additive	—	—	—	0.78 (0.61-0.99)	0.043

OR: odds ratio; 95% CI: 95% confidence interval; HWE p ≤ 0.05 is excluded.

Men are reported to be more common than women, to study the association between IL-4 SNPs and RCC based on gender, the study subjects were classified into two groups based on gender. The risk associated with IL-4 genotypes was calculated in each group. No significant association was found between females and IL-4 subjects in Table 4. However, we found four SNPs were associated with RCC risk in male (rs2243250(C), OR = 0.65, 95%CI = 0.46-0.90, P = 0.01; rs2243267(G), OR = 0.70, 95%CI = 0.50-0.97, P = 0.034; rs2243270(A), OR = 0.70, 95%CI = 0.50-0.97, P = 0.034; rs2243289(A), OR = 0.71, 95%CI = 0.51-0.99, P = 0.04). And Association between SNPs genotypes and RCC risk under different genotypic models in Supplementary Tables 2 and 3. In this research, we further evaluated age, smoking status, drinking status stratification and the risk of the IL-4 gene polymorphism of RCC. In Table 5, there was no significant correlation between age group > 55 and IL-4 polymorphism.

**Table 4:** Basic information of candidate SNPs in male and female

SNPs	Gene	Allele (A/B)	Male					Female				
			MAF (case)	MAF (control)	HWE	P	OR (95%CI)	MAF (case)	MAF (control)	HWE	P	OR (95%CI)
rs2243250	IL4	C/T	0.169	0.24	0.236	0.01	0.65	0.258	0.253	0.352	0.894	1.03
							(0.46-0.90)					(0.69-1.52)
rs2227284	IL4	G/T	0.13	0.174	0.05	0.074	0.71	0.157	0.169	0.803	0.696	0.91
							(0.49-1.03)					(0.57-1.45)
rs2243267	IL4	G/C	0.174	0.232	0.388	0.034	0.7	0.258	0.25	0.569	0.841	1.04
							(0.50-0.97)					(0.70-1.54)
rs2243270	IL4	A/G	0.174	0.232	0.388	0.034	0.7	0.258	0.25	0.569	0.841	1.04
							(0.50-0.97)					(0.70-1.54)
rs2243283	IL4	G/C	0.177	0.195	0.119	0.492	0.89	0.116	0.172	0.612	0.077	0.63
							(0.63-1.25)					(0.38-1.05)
rs2243289	IL4	A/G	0.174	0.23	0.49	0.04	0.71	0.263	0.24	0.33	0.545	1.13
							(0.51-0.99)					(0.76-1.67)

**Table 5:** The association between SNPs and age, smoking, drinking status analysis of CC patients

SNP	Allel(A/B)	Age>55		Age<55		Nosmoking		Smoking		Drinking		Nodrinking	
		OR(95%CI)	P	OR(95%CI)	P	OR(95%CI)	P	OR(95%CI)	P	OR(95%CI)	P	OR(95%CI)	P
rs2243250	C/T	0.91(0.64-1.29)	0.582	0.63(0.43-0.92)	0.016	0.67(0.44-1.02)	0.061	0.84(0.61-1.15)	0.269	0.49(0.27-0.91)	0.022	0.81(0.61-1.09)	0.163
rs2227284	G/T	0.89(0.59-1.34)	0.584	0.67(0.43-1.03)	0.064	0.60(0.37-0.96)	0.034	0.92(0.64-1.33)	0.652	0.45(0.22-0.95)	0.033	0.85(0.61-1.19)	0.342
rs2243267	G/C	0.97(0.68-1.38)	0.849	0.65(0.45-0.95)	0.024	0.74(0.49-1.11)	0.145	0.86(0.62-1.18)	0.351	0.50(0.27-0.93)	0.026	0.87(0.65-1.16)	0.330
rs2243270	A/G	0.97(0.68-1.38)	0.849	0.65(0.45-0.95)	0.024	0.74(0.49-1.11)	0.145	0.86(0.62-1.18)	0.351	0.50(0.27-0.93)	0.026	1.15(0.86-1.54)	0.330
rs2243283	G/C	0.76(0.50-1.15)	0.19	0.92(0.63-1.35)	0.678	0.93(0.60-1.44)	0.734	0.75(0.52-1.08)	0.117	0.81(0.46-1.44)	0.475	1.17(0.86-1.54)	0.339
rs2243289	A/G	0.98(0.69-1.40)	0.914	0.69(0.48-1.01)	0.056	0.72(0.47-1.08)	0.113	0.93(0.68-1.28)	0.663	0.52(0.28-0.96)	0.034	1.11(0.83-1.48)	0.486

But three SNPs (rs2243250, rs2243267, rs2243270) have significant association was found. Further analysis based on smoking status showed no significant association with smoking polymorphism, however, between the subjects of both nonsmoking groups and IL-4 rs2227284 (OR=0.60, 95%CI= 0.37-0.96, P=0.034) polymorphism was associated with RCC risk. A stratified model for the analysis of drinking status show no significant association association for both the nondrinking status polymorphisms, on the contrary, four SNPs (rs2243250, rs2227284, rs2243267, rs2243270) polymorphisms were found obviously decrease RCC risk in drinking people.

**Figure 1:**
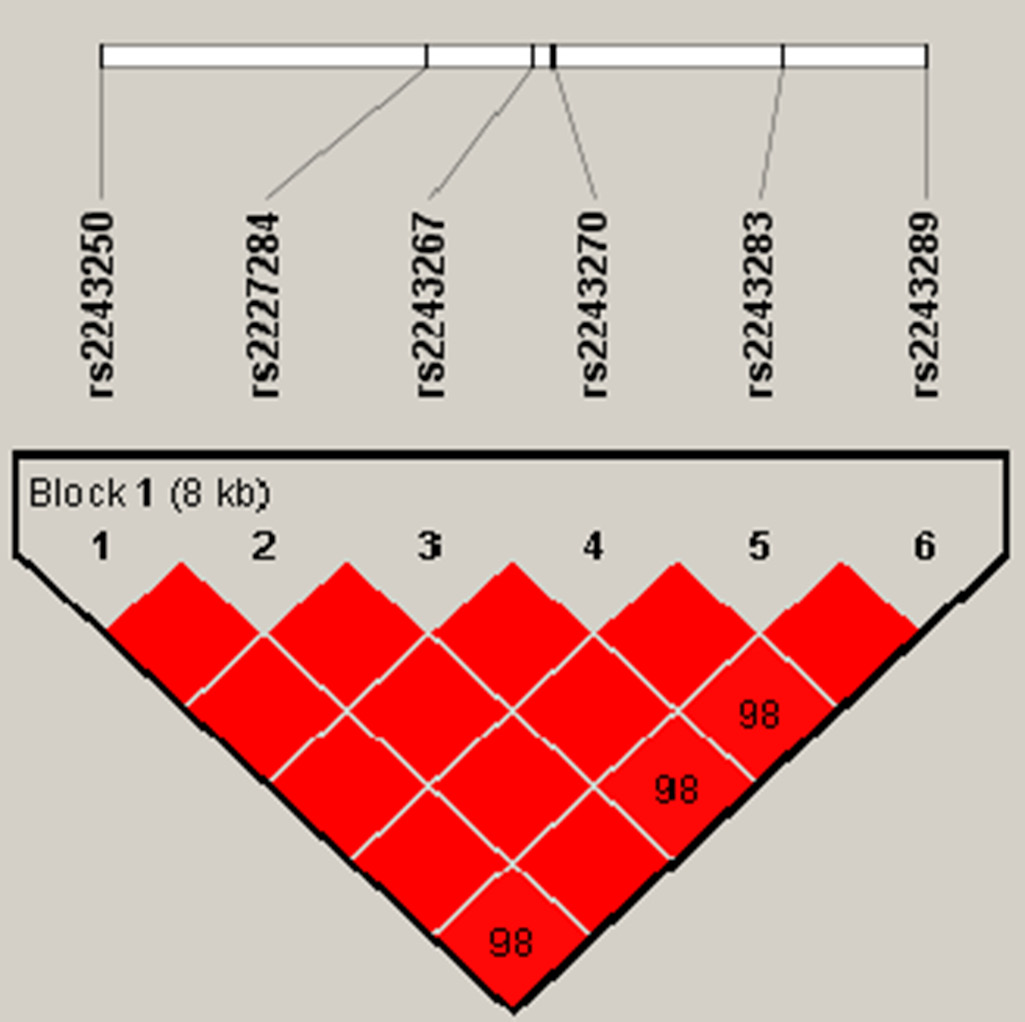
Haplotype block map for the IL-4SNPs genotyped in this study The LD between two SNPs is indicated by standardized D´(red boxes).

We futher analysed the LD and haplotype of the SNPs. One block was detected in studied IL-4 SNPs (Figure 1), IL-4 haplotype and the risk of RCC were listed in Table 6. Haplotype estimation analysis showed that the haplotype have a risk reduction in the risk of RCC was found in the rs2243250C/rs2227284G/ rs2243267G/rs2243270A/rs2243283C/ rs2243289A(CG GACA) genotypes compared with the TTCGCG genotype(Total, OR=0.73,95%CI=0.54-0.98;P=0.034; Male, OR=0.59 95%CI=0.39 - 0.90, P=0.014). Stratified analysis based on gender haplotype analyses in Supplementary Table 4.

**Table 6:** Haplotype frequencies and their associations with RCC risk in total

	rs2243250	rs2227284	rs2243267	rs2243270	rs2243283	rs2243289	Freq	OR (95% CI)	P
1	T	T	C	G	C	G	0.5937	1	—
2	T	T	C	G	G	G	0.1742	0.76 (0.58 - 1.01)	0.058
3	C	G	G	A	C	A	0.1585	0.73 (0.54 - 0.98)	0.034
4	C	T	G	A	C	A	0.0597	0.88 (0.57 - 1.37)	0.570

## DISCUSSION

In this study, we investigated whether the IL-4 gene polymorphism is associated with the risk of developing RCC in a case-control study. The obtained data illustrated a decreased RCC risk of individual carrying the “C” allel in rs2243250. Meanwhile, stratified analysis based on the age, gender, smoking status, drinking status revealed no significant association with RCC in age>55, female, smoking and nodrinking. However, for age<55, male, nonsmoking, and drinking status groups the SNPs polymorphisms were found obviously associated with RCC. We investigated the relationship between IL-4 gene polymorphism and RCC risk in Chinese population.

Currently, novel genetic and molecular predictors for the successful preventions and early diagnosis of cancers are needed [10]. Recent genetic studies have reported that many SNPs in cytokine genes are found are associated with a variety of diseases, including cancer [11]. Genetic polymorphism in cytokine genes was associated with the risk of development of certain types of cancer. Proinflammatory cytokines promote tumor development, and there is scientific evidence suggesting chronic inflammation can lead to the initiation and progression of cancer in a genetic controlled fashion [12].

It is found that there are lymphocytes, dendritic cells and macrophages infiltration in renal cell carcinoma, which suggests the immunogenicity of renal cell carcinoma [13, 14]. IL-4 is an anti-inflammatory immunosuppressive cytokine, which plays an important role in tumorigenesis and autoimmunity [15]. It is plays a central role to regulate the differentiation of antigen-simulated naive T cells, and then causes these T cells to produce cytokines, such as IL-10 and IL-14, or on the other hand, suppresses CD4^+^ T cells to secrete IFN-γ [16]. Hence, IL-4 has been seen as the focus of controversy in recent years due to the indeterminate biologic functions on cancers. However, variants in IL-4 and genes may effect on IL-4 signal transduction by change promoter activity of IL-4 gene and conformation of IL-4R protein directly or indirectly [17, 18]. Early studies suggested that IL-4 could help other cytokines or drugs to remove and suppress growth of tumor cells [19, 20]. Newly, studies have found that personality carrying the T allele of rs2243250 remarkably decreased the risk of colorectal cancer and gastric cancer, possibly by enhancing the Th2 responsed [21]. But, in the present study, we found that individuals carrying C alleles were associated with a significantly lower risk of developing RCC compared with the T allele.

The differences can be explained by the different mechanisms suggesting that different mechanisms of tumor development and the role of IL-4 genes in different tumors may be different. Thus, increased production of IL-4 may inhibit the proliferation of RCC in the early stages of the disease, but may cause Th2 cells to deviate and prevent immune surveillance of Th1 cells in disease progression. In addition, although IL-4 exhibited a proliferation inhibition effects *in vitro* of RCC [22, 23], the clinical use of IL-4 as an administrated agent failed to inhibit the progression of RCC and other cancers [24, 25]. Previously, Rosenwasser et al. [26] described that T allele of rs2243250 in IL-4 gene was associated with the increased expression of IL-4 *in vitro*. But IL-4 could not suppress the progression of RCC [24], even exhibit a possible “positive” activity of tumor progression.

Epidemiologic studies revealed a strong connection between smoking and drinking risk factors causes of RCC. Smoking caused by RCC, which may be caused by cigarette smoking induced immune suppression, so that RCC escape immune surveillance, thereby promoting the occurrence and development of RCC [27]. Studies have reported that between alcohol consumption and risk of RCC has a dose-dependent relationship, namely alcohol consumption to achieve a certain degree of risk exists only a small amount of alcohol, but can play the role of is beneficial to the body [28], this can be a very good explanation to our other hierarchical analysis results, the relative risk of drinkers for non-drinkers carrying the variant genotype and higher risk of RCC. These risk factors and genetic factors interact to affect the susceptibility of RCC. However, due to the limitations of data collection, the study of environmental factors is limited to smoking, drinking and other factors, so it is not able to completely analyze the interaction of gene environment. Therefore, it is necessary to carry out a complete analysis of the gene environment interaction, and to verify the results of the study.

In summary, we presented an association of IL-4 gene polymorphisms with RCC risk. And we found rs2243250(C/T) were associated with RCC risk. In addition we also found that for different gender, the risk of the individual is not the same. The loci we identified are likely to provide new insights into the etiology of RCC, especially differences in risk according to sex. These findings, after validation by larger studies, may help identify at risk populations for primary cancer prevention.

## MATERIALS AND METHODS

### Study population

The case-control study included 291 patients with RCC and 463 healthy controls. Cases of RCC were confirmed by pathology in an ongoing study that began recruiting in the Affiliated Hospital of Xizang Minzu University, Shaanxi, China. The patient had no previous history of any other type of cancer. Control group were randomly selected for the same period in the same hospital. The control group had no history of cancer. We used face-to-face interviews by trained interviewers to obtain information on demographic data and related factors. Collected potential confounders mainly included tobacco smoking, alcohol use, family history and previous diseases. All subjects were Han Chinese. After signing the informed consent, from each person, each donor donated 5 ml of blood for genomic DNA extraction. This study was approved by the institutional review board of the Ethics Committee of Xizang Minzu University.

### Selection of single nucleotide polymorphisms and genotyping

In the study, six SNPs in IL-4 were selected from DbSNP database (http://www.hapmap.org/index.html.en) and SNP Consortium database (http://snp.cshl.org/) for analysis and each had minor allele frequency (MAF) of > 5% in Chinese Han population. Whole blood were used the GoldMag-Mini Whole Blood Genomic DNA Purification Kit (GoldMag Co. Ltd. Xi’an City, China) extracted. We used a NanoDrop 2000 (Gene Company Limited) were measured DNA concentrations. We used Sequenom MassARRAY Assay Design 3.0 Software to design a Multiplexed SNP MassEXTEND assay [29]. Genotyping was performed on a Sequenom MassARRAY RS1000 platform using the manufacturer’s protocol. The PCR primers for each SNP are shown in Supplementary Table 1. Data management and analysis was performed using the Sequenom Typer 4.0 Software [29, 30].

### Statistical analysis

To ensure that the used controls were representative of the general population and to exclude the possibility of genotyping error, the deviation of the genotype frequencies of IL-4 SNPs in the controls from those expected under the Hardy-Weinberg equilibrium (HWE), was assessed using the χ^2^ test. Differences in the distributions of demographic characteristics, selected variables, and frequencies of genotypes of the IL-4 polymorphism between the cases and controls were evaluated by using the Student’s t-test or chi-square test. The association between IL-4 SNPs polymorphisms and the risk of RCC was determined by calculating the odds ratio (OR) at 95% confidence intervals (CI) for each polymorphism from unconditional logistic regression analysis with the adjustment for possible confounders. The risk of cancer will also be analyzed based on mean age, gender, smoking, alcohol consumption and calculating OR at 95% CI. P value of <0.05 was considered statistically significant.

## SUPPLEMENTARY MATERIALS TABLES




