# Supplementary material for: Associations between polymorphisms in the IL-4 gene and renal cell carcinoma in Chinese Han population
Source: Oncotarget. 2017 Jun 9;8(47):82078–84. doi: 10.18632/oncotarget.18427 (PMC5669871; doi:10.18632/oncotarget.18427)
Supplement: Supplementary file 1 [file oncotarget-08-82078-s001.pdf]

Associations between polymorphisms in the IL-4 gene and renal cell carcinoma in Chinese Han population

SUPPLEMENTARY MATERIALS

Supplementary Table 1: PCR primers

SNP	1st-PCR primer sequences	2st-PCR primer sequences	UEP sequences
rs2243250	ACGTTGGATGTAACAGGCAGACTCTCCTAC	ACGTTGGATGTGATACGACCTGTCCTTCTC	TAAACTTGGGAGAACATTGT
rs2227284	ACGTTGGATGGATGAAGGGTTTCTTGGGTG	ACGTTGGATGCATTATGGAACTCTCTGTAG	AGCTCTCTTTGGTAAATAGGAAAT
rs2243267	ACGTTGGATGTATAGTTTACTCACTGCCGC	ACGTTGGATGAGAAACGCATTGCACAGTGG	cccaCTATCGTGGCAGATTTTTG
rs2243270	ACGTTGGATGCAGTATCAACAGTTGACCCC	ACGTTGGATGACATTCACTCATCCCACCAG	CACCAGCCAGAGGTAACTA
rs2243283	ACGTTGGATGAAACAGTACTGACCATCGCC	ACGTTGGATGTGCTGACAGATCGGTTGTAG	tGGGGAGGAAAAGATGAC
rs2243289	ACGTTGGATGGGCTTGATCAAGTAGACAGG	ACGTTGGATGTCACAGGACAGGAATTCTGC	tatCTTGCATTGGTAAGCATTTGTC

Supplementary Table 2: Association between SNPs genotypes and RCC risk under different genotypic models in male

See Supplementary File 1

Supplementary Table 3: Association between SNPs genotypes and RCC risk under different genotypic models in Female

	Model	Genotype	Control	Case	OR (95% CI)	P-value
rs2069840	Codominant	C/C	169 (85.3%)	74 (75.5%)	1	0.048
		G/C	26 (13.1%)	23 (23.5%)	2.23 (1.17-4.23)	
		G/G	3 (1.5%)	1 (1%)	0.72 (0.07-7.08)	
	Dominant	C/C	169 (85.3%)	74 (75.5%)	1	0.024
		G/C-G/G	29 (14.7%)	24 (24.5%)	2.05 (1.10-3.82)	
	Recessive	C/C-G/C	195 (98.5%)	97 (99%)	1	0.67
		G/G	3 (1.5%)	1 (1%)	0.62 (0.06-6.10)	
	Log-additive	—	—	—	1.72 (0.98-3.00)	0.058

Supplementary Table 4: Haplotype frequencies and their associations with RCC risk in male

	rs2243250	rs2227284	rs2243267	rs2243270	rs2243283	rs2243289	Freq	OR (95% CI)	P-value
1	T	T	C	G	C	G	0.5984	1	—
2	T	T	C	G	G	G	0.1872	0.82 (0.56 - 1.21)	0.33
3	C	G	G	A	C	A	0.1543	0.59 (0.39 - 0.90)	0.014
4	C	T	G	A	C	A	0.0481	0.72 (0.34 - 1.51)	0.38
