# Supplementary material for: Associations between polymorphisms in the IL-4 gene and renal cell carcinoma in Chinese Han population
Source: Oncotarget. 2017 Jun 9;8(47):82078–84. doi: 10.18632/oncotarget.18427 (PMC5669871; doi:10.18632/oncotarget.18427)
Supplement: Supplementary file 2 [file oncotarget-08-82078-s002.docx]

**Supplementary Table 2: Association between SNPs genotypes and RCC risk under different genotypic models in male**

| **SNP** | **Model** | **Genotype** | **Control** | **Case** | **OR (95% CI)** | **P-value** |
| --- | --- | --- | --- | --- | --- | --- |
| rs2243250 | Codominant | T/T | 157 (59.2%) | 133 (69.3%) | 1 | 0.036 |
|  |  | C/T | 89 (33.6%) | 53 (27.6%) | 0.64 (0.40-1.01) |  |
|  |  | C/C | 19 (7.2%) | 6 (3.1%) | 0.37 (0.13-1.04) |  |
|  | Dominant | T/T | 157 (59.2%) | 133 (69.3%) | 1 | 0.018 |
|  |  | C/T-C/C | 108 (40.8%) | 59 (30.7%) | 0.59 (0.38-0.92) |  |
|  | Recessive | T/T-C/T | 246 (92.8%) | 186 (96.9%) | 1 | 0.089 |
|  |  | C/C | 19 (7.2%) | 6 (3.1%) | 0.43 (0.15-1.19) |  |
|  | Log-additive | --- | --- | --- | 0.62 (0.43-0.90) | 0.01 |
| rs2227284 | Codominant | T/T | 186 (70.2%) | 146 (76%) | 1 | 0.09 |
|  |  | G/T | 66 (24.9%) | 42 (21.9%) | 0.71 (0.43-1.16) |  |
|  |  | G/G | 13 (4.9%) | 4 (2.1%) | 0.34 (0.10-1.15) |  |
|  | Dominant | T/T | 186 (70.2%) | 146 (76%) | 1 | 0.064 |
|  |  | G/T-G/G | 79 (29.8%) | 46 (24%) | 0.64 (0.40-1.03) |  |
|  | Recessive | T/T-G/T | 252 (95.1%) | 188 (97.9%) | 1 | 0.089 |
|  |  | G/G | 13 (4.9%) | 4 (2.1%) | 0.37 (0.11-1.25) |  |
|  | Log-additive | --- | --- | --- | 0.65 (0.44-0.97) | 0.033 |
| rs2243267 | Codominant | C/C | 159 (60%) | 131 (68.2%) | 1 | 0.091 |
|  |  | G/C | 89 (33.6%) | 55 (28.6%) | 0.69 (0.44-1.09) |  |
|  |  | G/G | 17 (6.4%) | 6 (3.1%) | 0.42 (0.15-1.18) |  |
|  | Dominant | C/C | 159 (60%) | 131 (68.2%) | 1 | 0.048 |
|  |  | G/C-G/G | 106 (40%) | 61 (31.8%) | 0.64 (0.42-1.00) |  |
|  | Recessive | C/C-G/C | 248 (93.6%) | 186 (96.9%) | 1 | 0.14 |
|  |  | G/G | 17 (6.4%) | 6 (3.1%) | 0.47 (0.17-1.32) |  |
|  | Log-additive | --- | --- | --- | 0.67 (0.46-0.97) | 0.029 |
|  | Codominant | G/G | 159 (60%) | 131 (68.2%) | 1 | 0.091 |
| rs2243270 |  | G/A | 89 (33.6%) | 55 (28.6%) | 0.69 (0.44-1.09) |  |
|  |  | A/A | 17 (6.4%) | 6 (3.1%) | 0.42 (0.15-1.18) |  |
|  | Dominant | G/G | 159 (60%) | 131 (68.2%) | 1 | 0.048 |
|  |  | G/A-A/A | 106 (40%) | 61 (31.8%) | 0.64 (0.42-1.00) |  |
|  | Recessive | G/G-G/A | 248 (93.6%) | 186 (96.9%) | 1 | 0.14 |
|  |  | A/A | 17 (6.4%) | 6 (3.1%) | 0.47 (0.17-1.32) |  |
|  | Log-additive | --- | --- | --- | 0.67 (0.46-0.97) | 0.029 |
|  | Codominant | G/G | 159 (60%) | 131 (68.2%) | 1 | 0.07 |
| rs2243289 |  | G/A | 90 (34%) | 55 (28.6%) | 0.66 (0.41-1.04) |  |
|  |  | A/A | 16 (6%) | 6 (3.1%) | 0.42 (0.15-1.20) |  |
|  | Dominant | G/G | 159 (60%) | 131 (68.2%) | 1 | 0.031 |
|  |  | G/A-A/A | 106 (40%) | 61 (31.8%) | 0.62 (0.40-0.96) |  |
|  | Recessive | G/G-G/A | 249 (94%) | 186 (96.9%) | 1 | 0.16 |
|  |  | A/A | 16 (6%) | 6 (3.1%) | 0.48 (0.17-1.36) |  |
|  | Log-additive | --- | --- | --- | 0.65 (0.45-0.94) | 0.021 |
